# An exploration of Early Childhood Education students’ knowledge and preparation to facilitate physical activity for preschoolers: a cross-sectional study

**DOI:** 10.1186/1471-2458-14-727

**Published:** 2014-07-18

**Authors:** Olivia JM Martyniuk, Patricia Tucker

**Affiliations:** 1 Health and Rehabilitation Sciences Program, Faculty of Health Sciences, The University of Western Ontario, 1151 Richmond St, London, ON N6A 3K7, Canada; 2 School of Occupational Therapy, University of Western Ontario, 1201 Western Road, London, Ontario N6G 1H1, Canada

**Keywords:** Physical activity, Early childhood education students, Training, Preschool-aged children

## Abstract

**Background:**

Early childhood educators play an important role in influencing preschoolers’ physical activity levels. The current study sought to explore Early Childhood Education (ECE) students’ physical activity-related knowledge and educational experience during their formal training in Ontario.

**Methods:**

A total of 1,113 ECE students from 20 Ontario Colleges completed the study survey (online or on paper), which examined students’ physical activity course content; awareness of physical activity guidelines; understanding of health-related benefits of physical activity; self-efficacy to facilitate physical activity for preschoolers; self-reported physical activity levels; as well as physical activity-related resource needs. Descriptive statistics and independent samples t-tests were used to analyze the quantitative findings.

**Results:**

Survey results identified that 72.1% of ECE students had not completed any physical activity/physical education specific courses, while only 28.7% were familiar with, and 2.0% accurately reported, the Canadian Physical Activity Guidelines for the Early Years. Only 10.5% of ECE students reported personal physical activity behaviors consistent with national recommendations for adults (150 minutes/week). ECE students’ mean overall task self-efficacy to facilitate physical activity was 7.37 (*SD* = 1.64). Self-efficacy was significantly higher (*p* < .05) when students had taken one or more courses devoted to physical activity/physical education, as well as when students engaged in sufficient physical activity to meet the national guidelines for adults (*p* < .05).

**Conclusions:**

The results indicate that the current ECE college curriculum represents an excellent opportunity to provide future childcare providers with enriched physical activity-related training and support, such as physical activity guidelines, workshops, and new ideas for activities. Emphasizing the health benefits of physical activity for adults might be important in light of ECE students’ low self-reported physical activity levels.

## Background

Physical activity is a modifiable lifestyle behavior linked with healthy body weight [1], and reduced risk of chronic health conditions [2,3], most notably childhood obesity [4,5]. Children obtain many health benefits from engaging in regular physical activity including improvements in their social, cognitive, psychological, as well as physiological development [3,6-10]. Canadian guidelines suggest that preschool-aged children should accumulate a minimum of 180 minutes of physical activity each day and minimize sedentary time in order to achieve health benefits and develop fundamental movement skills [8,11]. Moreover, establishing physical activity as a habitual behavior early in a child’s life can foster an active adult lifestyle [12] and act as an important piece of the obesity prevention strategy. Such preventative measures are critical as preschool-aged children (aged 2.5-5 years) have not been exempt from the current obesity epidemic; 15.2% and 6.3% of Canadian preschoolers are classified as overweight or obese, respectively [13]. This rate has been echoed internationally, with 16.0% and 5.0%, 21.2% and 10.4%, and 15.2% and 5.5% of preschoolers being classified as overweight or obese in the United Kingdom, the US, and Australia, respectively [14-16]. These findings are particularly disconcerting as obese children are more likely to become obese adolescents [17], and are therefore, more susceptible to experiencing health complications later in life [18,19].

Although the childcare environment (e.g., center-based or home-based care) has been recognized as an ideal venue to promote physical activity for preschoolers [20,21], children enrolled in these facilities typically exhibit low levels of physical activity [2,22-27]. In recent objective studies in childcare, preschoolers were found to spend between 1.54 minutes [27] and 1.76 minutes [26] per hour participating in moderate-to-vigorous physical activity (MVPA). These findings are particularly concerning in light of the high number of children attending childcare (i.e., nearly 80% of children with working or studying mothers are enrolled in some form of childcare and typically average 29 hours per week in care; [28]). Childcare environments that are supportive of physical activity are congruent with higher levels of physical activity [20,29]. Consistent with recommendations in the “Best-Practice Guidelines for Physical Activity at Child Care” [30], researchers have confirmed the specific components of childcare centers that influence physical activity participation among preschoolers, identifying portable equipment [20,27], reduced sedentary opportunities [31], staff behaviors [27], and increased physical activity training/education for staff as significant [20]. Based on these findings, it is clear that childcare staff play an important role in supporting and facilitating physical activity participation in this environment.

Early childhood educators have a very impactful and challenging role in the childcare setting. In addition to caring for the children at their facility, early childhood educators are commonly responsible for developing and implementing the curriculum for the preschool class (or classes) for which they care [32,33]. The importance that early childhood educators place on physical activity and other health behaviors may be reflected in their choices for classroom programming [21]. In fact, preschool teachers have identified themselves as the primary “gatekeeper to the playground” in recognition of their control in the outdoor play environment [32].

As significant role models to the children in their care [2,24,34], early childhood educators’ can strongly influence children’s physical activity levels [20,27,34], play quality, and the extent of outdoor playtime [32]. In fact, their behaviors were predictive of children’s physical activity in Vanderloo’s cross-sectional study [27]. Higher levels of preschooler’s physical activity can be elicited if early childhood educators provide positive prompts [35,36], supportive equipment, participate in active play with the preschoolers (i.e., role modelling), and/or create opportunities for structured and unstructured activities [24,37]. On the other hand, researchers have shared concerns about early childhood educators’ enthusiasm towards and efforts in encouraging physical activity [35], their awareness of the role they play in preschoolers’ physical activity engagement, as well as their access to physical activity-related curricular materials [38]. Interestingly, factors such as self-efficacy and lack of co-worker engagement have been raised by early childhood educators as important barriers to facilitating physical activity within the early learning environment [21,32]. Regardless of any physical activity-regulating policies in place at a childcare facility, it is essential that all childcare workers be invested in children’s health by creating ample active opportunities.

Physical activity-related training and resources provided to early childhood educators appears to be limited [33,34,39,40]. Goldfield and colleagues [2] suggested that if early childhood education (ECE) students’ basic training included sufficient focus on physical activity, early childhood educators would be well positioned to effectively improve preschoolers’ physical activity behaviors. Likewise, Derscheid et al. [34] recommended that college classes or training for early childhood educators could inform ECE students of healthy nutrition and physical activity for preschoolers. Childcare providers have personally acknowledged their need for additional resources and training to implement physical activity with preschoolers [41,42], which should not go unrecognized [38,42].

Physical activity interventions targeting early childhood educators could produce substantial public health benefits considering the substantial number of children these providers influence [2]. This is especially true in light of a recent understanding that physical activity interventions appear to be the most successful when executed by early childhood educators in the childcare setting [43]. Copeland and colleagues [32] proposed that increased training and support for childcare providers was key to physical activity interventions, especially if the program helped improve childcare providers’ self-efficacy. While there are no known interventions that provide physical activity-related training to ECE students, an educational component is central to many of the successful physical activity interventions with practicing early childhood educators in childcare centers [44-46]. Providing rigorous physical activity-related education to the next generation of childcare providers as a whole could be a more efficient expenditure of future intervention funding.

In light of the above identification that early childhood educators play an important and influential role on preschoolers’ physical activity [20,34], coupled with the understanding that interventions which are provided in the childcare setting led by childcare staff are the most effective [43] highlights the appropriateness of intervening with this group. However, targeting ECE students prior to completion of their formal education may be a more cost-effective and pro-active approach to ensure that the next generation of childcare providers are informed, educated, and supportive of physical activity. To date, no Canadian or international research has investigated the physical activity-related training, knowledge, and education of ECE students within their formal education. As such, the primary purpose of the present study was to explore the physical activity-related knowledge and educational experience of ECE students currently studying at Ontario colleges. As a secondary objective, ECE students’ task self-efficacy, their own physical activity behaviors, and their perspectives regarding resources that would aid in encouraging physical activity participation among preschoolers in childcare were explored.

## Methods

### Study design

Using a cross-sectional design, all eligible English-speaking Ontario colleges that offered an ECE diploma program (*n* = 23) were invited to participate in the study. The appropriate contact (e.g., ECE program coordinator and/or the Dean/Chair of the program) at each college was contacted in February 2013, to invite their students’ participation. Of the colleges that chose to participate (*n* = 20), all ECE students enrolled in their diploma program were invited to participate in the survey. The program contact or college Research Ethics Board was responsible for indicating their preferred method of data collection: (a) researchers distributed the survey (in person) to the class during a classroom session; (b) a professor/ECE coordinator disseminated the survey (in person) during class time; or (c) the program coordinator sent an email to all students enrolled inviting them to participate in the survey online through SurveyMonkey^®^. Of the colleges that chose to use the online survey, the ECE program coordinator was asked to send an email invitation to students or use Blackboard (an online course curriculum website or equivalent) to post the invitation. One reminder email was sent 2 weeks after the initial invitation. The online survey link remained accessible to ECE students from February 2013 to June 2013.

For those schools which chose to administer the survey on paper, a date was arranged for the surveys to be distributed in person by the researcher, or the surveys were mailed to the college contact for distribution in class. Completion of the survey indicated consent to participate. Ethical approval was provided by the Health Sciences Research Ethics Board at the University of Western Ontario. The study protocol was also submitted to requesting colleges’ (*n* = 9) Research Ethics Boards for approval.

### Participants and data collection

Using the Horatio sample size calculator, at an alpha of 0.05, a total sample size of 388 individuals was required to detect a small effect size (*r*^*2*^ = .02) between groups (with a power of 0.80). To ensure a diverse sample was captured, all students enrolled in the ECE diploma program at an agreeing college (*n =* 20) were invited to participate in the study regardless of which type of program they were attending (e.g., full-time, part-time, accelerated, or fast-track) and which year of study they were in (e.g., year 1, year 2, or other). Of the colleges that chose to partake in the study, 9 opted for online data collection (individual student responses per school ranged from 5 to 53 participants), 9 selected the paper version (individual student responses per school ranged from 6 to 262 participants), and 2 used both options (class specific).

### Measures

Individuals who participated in the study completed a survey created specifically for this project by the researchers. Using 24 questions, the survey was designed to examine ECE students’ knowledge (e.g., awareness of the Canadian Physical Activity and Sedentary Behavior Guidelines [8]) and college training (e.g., physical activity curriculum courses and concepts covered during courses [adapted from the National Association for Sport and Physical Education] [47]) to support physical activity for preschoolers. Relying on Bandura’s [48] guide to help develop the self-efficacy section of the survey, ECE students’ self-efficacy levels were assessed using 5 items (e.g., confidence to create active opportunities) to generate an aggregated score for self-efficacy to facilitate physical activity. The survey also explored ECE students’ awareness of environmental influences on children’s physical activity levels (developed using the Environment and Policy Assessment and Observation tool [49] as well as several research studies) and their comprehension of their roles surrounding physical activity. Various academic research articles (e.g., studies that focused on physical activity in childcare) and physical activity-related documents [50,51] were also used to develop the survey and ensure the survey content and language used was consistent with the early childhood education field. Once created, the survey was reviewed by researchers with expertise in preschoolers’ physical activity behaviors and a variety of ECE trained professionals to ensure the language and questions were appropriate prior to administration. The survey finished with an optional, open-ended question allowing ECE students to declare any beneficial resources or knowledge that would help them to facilitate physical activity for preschoolers.

### Statistical analysis

Frequencies and/or means/standard deviations were run to examine participants’ demographic characteristics, the number of students who received physical activity-related training during course content, as well as how many were familiar with particular physical activity documents (e.g., the Canadian Physical Activity Guidelines for the Early Years). To analyze self-efficacy, an aggregated mean was calculated to represent each student’s overall confidence to facilitate physical activity. Intra-class correlation coefficients (ICCs) were run to verify the appropriateness of calculating an aggregated mean. Two independent samples t-tests were performed to compare ECE students’ overall self-efficacy ratings to facilitate physical activity when segregated into two groups. The first t-test compared ECE students’ confidence dependent on the completion of any physical activity/education courses during their college training (1 or more courses versus no courses). An Analysis of Covariance (ANCOVA) was run to verify whether year of study had an influence on self-efficacy scores. A second t-test was completed to evaluate the difference (if any) between ECE students’ confidence ratings based on their own physical activity behavior (i.e., meeting the Canadian adult physical activity guidelines or not meeting the guidelines). All analyses were run using SPSS (version 21).

For the open ended question, which offered qualitative feedback from participants, data analysis involved inductive content analysis to allow common themes to emerge from the participants’ comments [52]. To achieve this, the researchers relied on Miller and Crabtree’s [53] process to review the quotations and identify emerging themes, which were consistently mentioned by the ECE students. The four criteria (i.e., credibility, transferability, dependability, and confirmability) described by Lincoln and Guba [54] were used as a framework (when applicable), for ensuring the accuracy of the qualitative findings.

## Results

Overall, 229 ECE students completed the survey online, and 884 completed a paper survey, for a total sample of 1,113. Our response rate was 9.35% and 85.25%^a^ for online and paper, respectively. As data collection was not feasible at two schools during the winter 2013 semester (i.e., January to April 2013), 109 surveys were completed in May/June 2013. The mean age of participating ECE students was 23.92 ± 7.43 years; 96.9% were female. The majority of students were enrolled in the diploma program full-time (n = 1,055), with 52.1% of first-year and 44.0% second-year students. See Table 1 for complete participant demographic information.

**Table 1:** **ECE students’ demographic information and physical activity experience (*n*** **= 1113)**

**Participant characteristic**	***N***	***%***
Sex		
Male	32	2.90
Female	1079	96.90
Age		
19 years or less	312	28.10
20-29 years	605	54.30
30-39 years	94	8.40
40+	76	7.00
Ethnicity		
Caucasian	850	76.40
African Canadian	24	2.20
Aboriginal	32	2.90
Hispanic	25	2.20
Asian	90	8.10
Arab	18	1.60
Other	50	4.50
Enrollment status		
Full-time	1055	94.80
Part-time	55	4.90
Year of study		
1	580	52.10
2	490	44.00
Other (e.g., accelerated or intensive)	41	3.70
Accumulated minutes of MVPA during a typical week		
<30	222	19.90
30-59	286	25.70
60-89	232	20.80
90-119	149	13.40
120-149	100	9.00
150+	117	10.50
Number of physical activity or physical education courses completed		
No courses completed	802^a^	72.10
1 course	174^a^	15.60
2 courses	49^a^	4.40
3 or more courses	27^a^	2.40

^a^Response rates for paper surveys are based on the number of ECE students that the college granted access to (i.e., the proportion of students who completed the survey out of the total number of students in attendance from the participating classrooms) and not representative of the total amount of ECE students in the program.

*Note.* Column totals per section may not always match the total number of participants due to skipped questions; ECE = early childhood education; MVPA = moderate-to-vigorous physical activity.

### Participant’s physical activity experiences

The majority (91.6%) of ECE students indicated that physical activity was either ‘somewhat important’ or ‘very important’ in their own lives. Only 10.5% of ECE students reported physical activity behaviors consistent with the national guidelines of 150 minutes per week [8], summarized in Table 1.

### Physical activity-related education and training

During their college education, 802 ECE students (72.1%; Table 1) reported having completed no physical activity/physical education specific courses, with 174 ECE students (15.6%) having completed one course, such as “Children’s Health and Wellness” or “Music and Movement.” Of the mandatory course lessons in their college education, just over half of ECE students (66.5%; Table 2) reported that physical activity was discussed. Many ECE students recognized several health benefits associated with children participating in physical activity, such as increased coordination or enhanced brain function. While 82.0% of ECE students had studied the relationship between physical activity and childhood obesity during their ECE training, only 16.2%, 44.0%, and 45.9% reported having discussed the relationship between physical activity and cancer, heart disease, and type II diabetes, respectively.

**Table 2:** Physical activity course content during mandatory and/or elective early childhood education course lessons

	**Mandatory**		Elective*	
**Topic**	***N***	**%**	***N***	**%**
Physical education	421	37.80	131	11.80
Physical activity	740	66.50	167	15.00
Gross motor skills	929	83.50	164	14.70
Locomotor & non-locomotor skills	520	46.70	82	7.40
The F.I.T.T. Principle	101	9.10	57	5.10
No courses discussed these topics	91	8.20	367	33.00

*Note.* Column totals per section may not match the total number of participants due to skipped questions; F.I.T.T. = Frequency, Intensity, Time, and Type; *45.6% of students skipped the elective course question.

With regard to childcare and physical activity guidance documents, over the course of their ECE educational training, very few ECE students were familiar with the Canadian Physical Activity Guidelines for the Early Years (28.7%; Table 3). This is interesting, given that approximately one-quarter (23.4%) of ECE students believe that “most” Canadian preschoolers engage in sufficient levels of physical activity per day (as per the Canadian guidelines). However, only 2.0% of ECE students accurately reported the number of minutes Canadian preschoolers should be physically active per day (as per the guidelines). This translates into 61.7% of the students underestimating the duration of time that preschoolers should be active each day—which is a majority of the sample, considering 27.7% of students were not familiar with the document to answer this question. Only 6.8% of students correctly estimated the Sedentary Behavior Guidelines for this cohort.

**Table 3:** Early childhood education students’ familiarity with childcare and physical activity-related documents

**Guidance document**	***N***	***%***
The day nurseries act	1, 032	92.70
Active healthy kids Canada report card	181	16.30
Canadian physical activity guidelines for the early years	319	28.70
Canadian sedentary behaviour guidelines for the early years	93	8.40
I have never heard of any of these documents	31	2.80

*Note.* Column totals may not match the total number of participants due to skipped questions.

### ECE students’ views on the influences of preschoolers’ physical activity

A total of 94.7% of ECE students in the current study felt that physical activity was ‘very important’ for preschoolers. Most of these participants (ranging from 70.4% to 90.8%) concurred that early childhood educators themselves play a role in engaging preschoolers in physical activity by way of role modeling, providing equipment, creating structured and unstructured activities, and providing verbal prompts. However, students seemed less informed about the environmental influences on physical activity: only 53.5% of students indicated that teacher prompts are influential on physical activity, and only 62.0% and 63.0% recognized the impact of fixed and portable equipment, respectively.

### ECE students’ self-efficacy to instruct and facilitate physical activity for preschoolers

On a 10-point Likert scale assessing ECE students’ confidence to facilitate physical activity, their answers ranged from 1 to 10. Across the five task self-efficacy items measured, the ICC was 0.878 (with a 95% confidence interval [0.843, 0.903]). ECE students’ overall mean self-efficacy to facilitate physical activity was 7.37 (*SD =* 1.64). An independent samples t-test indicated that in comparison to students with no physical activity/education training (*n =*777^b^; mean = 7.29; *SD* = 1.65), ECE students who had completed one or more physical activity/education course (*n =* 244^b^) were significantly more confident (mean = 7.72; *SD* = 1.52) in their ability to facilitate physical activity for preschoolers (*t*[1019] = -3.63; *p* < .05).

With regard to ECE students own physical activity levels, results from the independent sample t-test suggest that students who were sufficiently active (i.e., met the Canadian Physical Activity Guidelines for Adults [8]; *n =* 114^b^) were more confident (mean = 8.00, *SD =* 1.48) to facilitate physical activity than their less active peers (*n =* 940^b^; mean = 7.29, *SD =* 1.64; *t*[1052] = -4.42; *p* < .05).

### Resources necessary to support physical activity

Only 5.0% of ECE students’ felt that no extra resources would be helpful to increase physical activity participation in the childcare environment, but the following were identified as beneficial: age/developmentally appropriate ideas for games/activities/exercises (84.5%); active opportunity ideas in various weather climates (75.8%); physical activity workshops or professional development programs (74.8%); instructional strategies to promote physical activity; and, guest physical activity instructors (56.7%). In addition to the resources listed within this question, ECE students’ also provided qualitative responses regarding beneficial resources or knowledge that would help them to facilitate physical activity for preschoolers. Emerging themes included: the creation of a course devoted to physical activity; physical activity-related workshops and/or professional development opportunities; as well as specific physical activity resources or guidelines. Please see Table 4 for quotes that support these findings.

**Table 4:** ECE students’ report of additional knowledge or resources beneficial to facilitating physical activity in preschoolers

**Theme**	**Example reflections**
New physical activity course	“Instating a mandatory ECE class that is solely based on physical activity.”
	“There should be one class based on physical activity for children because it is very important.”
	“Include a course within the ECE curriculum that strictly focuses on physical activities, health, and well-being.”
	“I definitely think that a course would be beneficial to early childhood educators in training regarding how to teach physical education to young students.”
Workshops/professional development	“Continued professional development on ways to promote physical activity within our children and ways to communicate healthy lifestyle to our families that we deal with.”
	“I think it’s important to have specific guidelines on training for gross motor activities and more workshops on it. I think that if we can inspire adults (teachers and parents alike) to be more active it will help in the classroom.”
	“Time built into courses for workshops.”
Resources and guidelines	“An online webpage that is specific with activities to develop certain areas of physical development such as cardio, muscular, flexibility, coordination. With a list of activities as well of how it’s helping that particular area of development.”
	“A physical activity website.”
	“List of websites that offer additional resources and videos to see children in action and more ways to help explain fitness to children so they will understand.”

*Note.* ECE = Early Childhood Education.

## Discussion

The purpose of the current study was to examine ECE students’ physical activity-related knowledge and educational experience during their college training. As a secondary objective, ECE students’ self-efficacy and self-reported physical activity behaviors were explored. This research represents the first Canadian or international study to explore ECE students’ knowledge and preparation to facilitate physical activity for preschoolers.

The majority of participating ECE students had not completed any specific physical activity/education courses during their college training. While it is possible that these students could go on to participate in training of this nature once they are working in the field (via professional development), past research has indicated that this may not actually be the case. Kim et al., reported that only 66.7% of home-care providers and 43.0% of center-based childcare providers had participated in physical activity training in the year prior [55]. Similarly in Australia, only 49.0% of preschool staff and 50.0% of childcare center staff had completed any physical activity training within the past year [56]. If physical activity-related training is this inconsistent for ECE students and practicing early childhood educators alike, implementing a mandatory physical activity course or increased course content as part of their college training may represent an excellent opportunity to improve the physical activity-related knowledge and education of all future childcare providers. This approach is supported by a recent study in Belgium which compared preschooler’s activity levels on days with allocated periods of teacher-led structured physical activity (versus days with no structured physical activity sessions). This study identified the effectiveness of teacher-led sessions at increasing activity levels (p < 0.001) while in care [57]. Interestingly, ECE students’ formal education in Belgium involves training on how to facilitate structured physical activities for children in their care [57], perhaps accounting for the above finding.

Although a number of students in the current study declared that physical activity is discussed during their mandatory course lessons, it is evident that further enhancements could be made to the type and extent of physical activity-related material instructed. While it seems important that ECE students be taught the national Physical Activity Guidelines for the Early Years (0–4 years), only 28.7% reported receiving this course content. Furthermore, only 2.0% accurately reported the appropriate minutes of physical activity per day [8] with many students underestimating the daily activity recommendations for preschoolers. This knowledge gap highlights that ECE students lack a comprehensive understanding of some valuable documents regarding preschoolers’ health behaviors that could be guiding their prospective practices and positively influencing their programming decisions in childcare. It is concerning to think that the individuals who are in a powerful position over many preschoolers during childcare hours are uninformed about the amount of time these children should spend being active each day for healthy growth and development.

Almost all of the ECE students in the present study were familiar with Ontario’s Day Nurseries Act [50]; however, this well-known document does not provide comprehensive legislation or policies on preschoolers’ daily physical activity during childcare hours (it mandates outdoor play time, not physical activity [58]). Therefore, introducing ECE students to the Canadian Physical Activity and Sedentary Behaviour Guidelines [8] could serve as excellent supplements to the Day Nurseries Act [50]. The Canadian guidelines are important for educating ECE students, given that the Ontario Day Nurseries Act has not been updated in 23 years, despite increases in childhood obesity, and consistent reports of low physical activity participation among preschoolers in childcare.

Based on their ECE training to date, ECE students’ overall task self-efficacy to facilitate physical activity was 7.37 (*SD =* 1.64). This finding suggests that the physical activity-related content that exists in the current curriculum may be providing ECE students with a reasonable amount of knowledge and skills to contribute to their self-efficacy, though room for improvement exists nevertheless. Based on our t-test results, ECE students with increased physical activity/education training appeared to have higher confidence to facilitate physical activity. Other research confirms that this connection between training and confidence would be likely. Simpson, Tucker, and van Zandvoort [59] found a similar relationship in their study of 58 physical education teachers: although not statistically significant, increased training may impact teachers’ confidence when teaching physical education. Given that childcare providers have previously reported feeling they have less knowledge and self-efficacy in physical activity provisioning [32,34,60], perhaps additional training could help to overcome this barrier [34]. Our recommendations align with those by Derscheid et al. [34] who suggest that physical activity college classes or training could have potential implications on the confidence of early childhood educators to facilitate physical activity for children in their care.

Another finding worthy of discussion is that only 10.5% of ECE students from the present study reported participation in weekly MVPA at a level that meets the Canadian Physical Activity Guidelines for Adults (≥150 minutes of MVPA per week; [8]). While these data were based on a self-report questionnaire, it could be problematic to have insufficiently active childcare workers, especially since 87.10% of participants had reported placement experience at a childcare center where they had an influence on children in their care. More physically active early childhood educators may have a positive influence in the childcare environment given the importance of modeling physical activity to children [60] and the value they place on this behavior [41]. A strong interest in physical activity could influence providers’ motivation to prioritize and model physical activity during preschool hours. The results of the present study support the notion that ECE students’ physical activity behaviors may influence their confidence to deliver physical activity in the workplace: ECE students were more confident in their abilities to facilitate physical activity when they were more active themselves. These findings align with Simpson et al. [59] indicating that a correlation exists between physical education teachers’ confidence to instruct physical activity and their personal activity behaviors. This result indicates that training should emphasize the value and importance of leading a physically active lifestyle, for both children and adults.

### Future implications

In terms of research implications, this study serves as an important platform for future physical activity research investigations or interventions with early childhood educators. Future research should consider a more in-depth account of ECE students’ perspectives on the desire for, and types of physical activity-related courses or content that could be added to the ECE curriculum. It would also be beneficial to obtain classroom professor’s perspectives on the current course content focused on physical activity within the current ECE curriculum, given it is possible that course content was taught, but the ECE students in this study missed the class or forgot about that material when answering the survey. Finally, the current study could be broadened to a national or international level to see if any differences exist between institutions across the globe regarding physical activity training in the ECE field and/or ECE students’ physical activity-related knowledge.

### Strengths and limitations

This study makes an important contribution to the literature on preschoolers’ physical activity in the childcare environment as it is the first to gather an understanding of the physical activity-related training of ECE students. The current study heightens awareness of certain physical activity-related gaps in ECE students’ knowledge and curriculum that might be considered in future revisions to the ECE curriculum. While the present study offers a unique contribution, it is not without limitations. Although the total sample size was large, it would have been ideal if all of the participants had answered the survey at the end of their graduating year. More students completed the survey on paper than online (perhaps due to the presence of a researcher/professor/teacher assistant), and the number of responses received was disproportionate to the number of students attending each school (e.g., one school had 6 students participate, while another school had 363 students participate), which potentially skews the data and threatens our external validity. Despite this, there was an excellent overall scope and sample size to garner an understanding of ECE students learning experiences across Ontario. In addition, some of our smaller response rates may be explained by the fact that we were unable to arrange data collection at some colleges until the final few weeks of the semester. The possibility for volunteer bias and social desirability bias from the participants was not absent. While it was made clear that the survey was voluntary and anonymous, students completing the paper survey may have felt obliged to participate when the researcher, professor, and/or college staff member was present in the room during survey distribution. Given that no validated, reliable instrument was found to fulfill the purpose of this study, the measurement method used for the present study was created by the researchers and was not formally validated or tested for reliability. While this may affect the internal validity of the current study, both researchers and experts in the field reviewed the survey to ensure face validity.

## Conclusions

Based on the specific findings from the current study, ECE students require more supportive training on the relationship between physical activity and health outcomes; on accessing activity guidelines for the early years; as well as resources that are desired by ECE students, such as age/developmentally-appropriate physical activities and active opportunities ideas in various weather climates. Moreover, by providing this knowledge proactively during early childhood educators’ formal training, it is possible that, once they enter the workforce, these childcare providers will be champions for physical activity being provided to the preschoolers in their care. Given that early childhood educators are employed at a variety of facilities (i.e., center-based childcares, home-based daycares, early learning programs, or before and school programs) which may lack enforced regulations for physical activity-related practices and policies, it is crucial that early childhood educators be targeted as students to ensure that they enter the workforce with sufficient physical activity-related knowledge, skills, and training to confidently structure physical activity within their childcare programming. Relying solely on the creation of physical activity-related provincial policies, or physical activity workshops/interventions to support current early childhood educators with increased skills and resources significantly reduces the overall reach and impact of these initiatives. A greater number of children would benefit if providers were equipped with physical activity-related knowledge and skills prior to entering the workforce. The ECE curriculum presents a unique opportunity to support these individuals with such comprehensive physical activity knowledge and training.

## Endnotes

^a^Response rates for paper surveys are based on the number of ECE students that the college granted access to (i.e., the proportion of students who completed the survey out of the total number of students in attendance from the participating classrooms) and not representative of the total amount of ECE students in the program.

^b^Group sizes represent students who answered both the physical activity training and self-efficacy questions; these numbers differ from relative data presented in Table 1.

## Abbreviations

CSEP: Canadian society for exercise physiology; ECE: Early childhood education; ECEs: Early childhood educators; EPAO: Environment and policy assessment and observation; MVPA: Moderate-to-vigorous physical activity; PA: Physical activity.

## Competing interests

The authors declare that they have no competing interests.

## Authors’ contributions

OM & PT developed the study design, methodology, and survey tool for this project. OM & PT were responsible for recruiting the Ontario Colleges and coordinating data collection. OM distributed surveys at eligible colleges. OM & PT performed statistical analysis. OM drafted the original manuscript and PT revised the manuscript for important modifications. Both authors read and approved the final manuscript.

## Pre-publication history

The pre-publication history for this paper can be accessed here:

http://www.biomedcentral.com/1471-2458/14/727/prepub
